# Purslane-induced oxalate nephropathy: case report and literature review

**DOI:** 10.1186/s12882-023-03236-9

**Published:** 2023-07-13

**Authors:** Xiangtuo Wang, Xiaoyan Zhang, Liyuan Wang, Ruiying Zhang, Yingxuan Zhang, Lei Cao

**Affiliations:** Department of Nephrology, Harrison International Peace Hospital, Renmin Road, Hengshui, 053000 Hebei Province People’s Republic of China

**Keywords:** Oxalate nephropathy, *Portulaca oleracea*, Purslane, Hyperoxaluria, Acute kidney injury, Case report

## Abstract

**Background:**

The kidney is particularly vulnerable to toxins due to its abundant blood supply, active tubular reabsorption, and medullary interstitial concentration. Currently, calcium phosphate-induced and calcium oxalate-induced nephropathies are the most common crystalline nephropathies. Hyperoxaluria may lead to kidney stones and progressive kidney disease due to calcium oxalate deposition leading to oxalate nephropathy. Hyperoxaluria can be primary or secondary. Primary hyperoxaluria is an autosomal recessive disease that usually develops in childhood, whereas secondary hyperoxaluria is observed following excessive oxalate intake or reduced excretion, with no difference in age of onset. Oxalate nephropathy may be overlooked, and the diagnosis is often delayed or missed owning to the physician’s inadequate awareness of its etiology and pathogenesis. Herein, we discuss the pathogenesis of hyperoxaluria with two case reports, and our report may be helpful to make appropriate treatment plans in clinical settings in the future.

**Case presentation:**

We report two cases of acute kidney injury, which were considered to be due to oxalate nephropathy in the setting of purslane (*portulaca oleracea*) ingestion. The two patients were elderly and presented with oliguria, nausea, vomiting, and clinical manifestations of acute kidney injury requiring renal replacement therapy. One patient underwent an ultrasound-guided renal biopsy, which showed acute tubulointerstitial injury and partial tubular oxalate deposition. Both patients underwent hemodialysis and were discharged following improvement in creatinine levels.

**Conclusions:**

Our report illustrates two cases of acute oxalate nephropathy in the setting of high dietary consumption of purslane. If a renal biopsy shows calcium oxalate crystals and acute tubular injury, oxalate nephropathy should be considered and the secondary causes of hyperoxaluria should be eliminated.

## Background

**Fig. 1:**
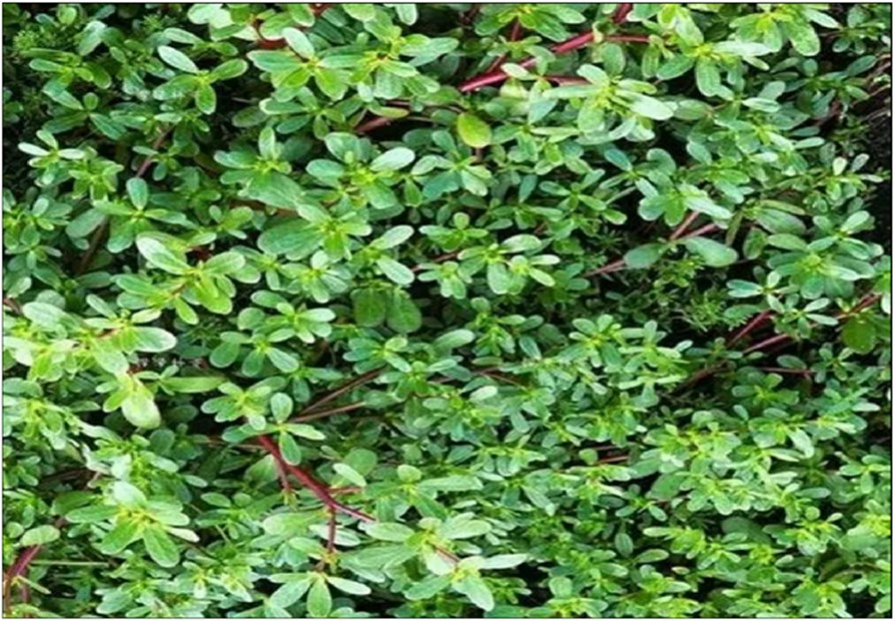
Purslane is widely grown in the land

Toxic nephropathy is an important cause of renal injury, such as herbal nephropathy, and the mechanisms underlying renal poisoning include direct damage to tubular cells, renal ischemia, crystalluria, and allergic reactions [1]. Purslane is known botanically as *Portulaca oleracea* and is an edible medicinal herb naturally abundant in grasslands and fields (Fig. 1) [2]. In Chinese traditional medicine, it is known as Ma Chi Xian and is also well known in European traditional medicine. Purslane leaves contain many phytochemicals with multiple medicinal properties, including flavonoids, alkaloids, polysaccharides, polyunsaturated fatty acids, sterols, essential dietary minerals, vitamins, and organic acids like oxalate [3]. Despite these beneficial effects, purslane has a high oxalate content and can cause hyperoxaluria [4]. Diet-induced oxalate nephropathy is rare and no specific treatment guidelines exist.

Herein, we report two cases of acute kidney injury (AKI) associated with the excessive consumption of purslane, one of which was confirmed as oxalate nephropathy by renal pathology and recovered rapidly after renal replacement therapy.

## Case presentation

### Case 1

**Fig. 2:**
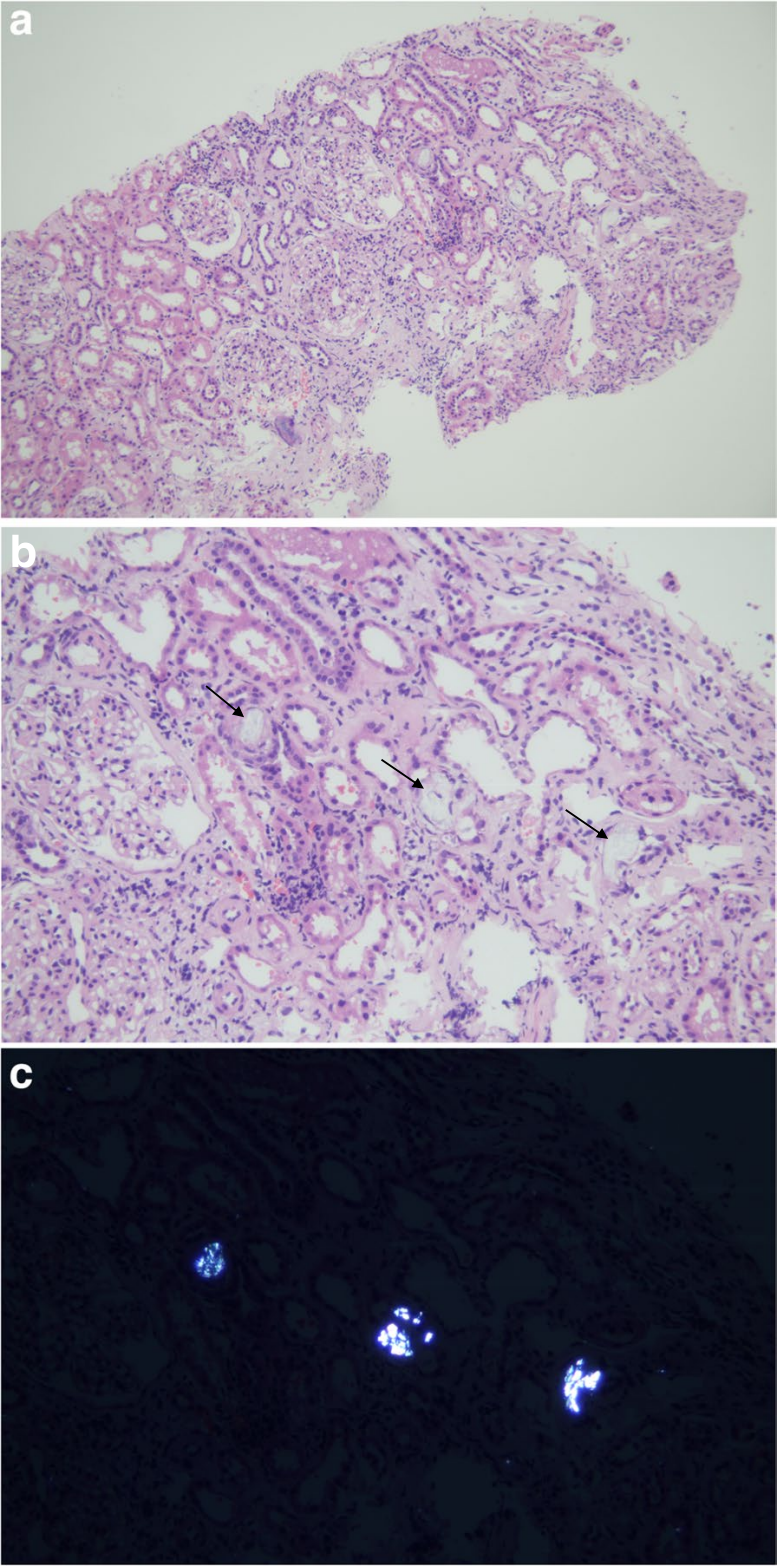
Renal biopsy showing oxalate crystallization in Case 1. **a** Kidney biopsy tissue under hematoxylin and eosin staining (H&E, × 100). **b** Translucent calcium oxalate crystals in the renal tubules (black arrows) under light microscopy (H&E, × 200). **c** The same field as b is shown under the polarized light microscope, and the birefringent crystal is visible (× 200)

A 58-year-old woman with diabetes mellitus (DM) was sent to our facility because of elevated creatinine level. Her DM was controlled by metformin and glipizide. She had a history of hypertension for more than 10 years, with the highest recorded blood pressure of 160/100 mmHg with oral sustained-release nifedipine tablets. She had no history of kidney disease, use of nonsteroidal anti-inflammatory drugs, or gastrointestinal surgery. The patient’s baseline serum creatinine was unknown and she did not take calcium supplements or herbal medicines. She had consumed approximately 0.75 kg of purslane stir-fry from her sister's vegetable garden in one meal seven days before admission, followed by vomiting, diarrhea, and oliguria. ​It was not the first time she had ingested purslane. Upon admission, her temperature was 35.6 °C, blood pressure was 120/57 mmHg, and laboratory data showed the following results: serum creatinine, 701.2 μmol/L (reference range, 41–81 μmol/L); blood glucose, 1.7 mmol/L; lactic acid, 12.4 mmol/L (reference range, 0.5–2.0 mmol/L); white blood cells, 11.27 × 10^9^/L; hemoglobin, 138 g/L; platelets, 430 × 10^9^/L; complement C3, 1.05 g/L (reference range, 0.79–1.52 g/L); complement C4, 0.43 g/L (reference range, 0.12–0.36 g/L); immunoglobulin A, 3.74 g/L (reference range, 0.69–3.82 g/L); immunoglobulin G, 9.62 g/L (reference range, 7.23–16.85 g/L); immunoglobulin M, 0.20 g/L (reference range, 0.63–2.77 g/L); parathyroid hormone, 83.3 pg/mL, and hemoglobin A1c, 10.6%. Urine test results were as follows: urine red blood cells, 6 /uL; urine protein, 1 + ; urine glucose, 1 + ; crystallization, (-); urine specific gravity, 1.007; 24 h proteinuria, 748 mg/24 h; urinary microalbumin, 106.1 mg/L (0–30); urinary beta2-microglobulin, 1.46 mg/L (0–0.24); urinary alpha1-microglobulin, 77.02 mg/L (0–18); urinary microalbumin/creatinine ratio, 234.8 mg/g. cr (0–30). Myeloperoxidase- antineutrophil cytoplasmic antibody (MPO-ANCA), proteinase 3 antineutrophil cytoplasmic autoantibody (PR3-ANCA), and anti-glomerular basement membrane (anti-GBM) antibody levels were negative. Ultrasonography showed that the left kidney was 109 × 55 × 65 mm in size, with a cortical thickness of 28 mm, whereas the right kidney was 102 × 56 × 61 mm, with a cortical thickness of 24 mm, without obstruction. Continuous renal replacement therapy was administered for 14 h on the day of admission with a urine volume of 350 mL/24 h. On the third day of hospitalization, urine volume increased to 1250 mL. She received alternate-day hemodialysis through a temporary femoral venous dialysis catheter for 10 days. The serum creatinine at the time of the last dialysis was 307.5 μmol/L on the 12^th^ day of admission. Initially, the patient refused a kidney biopsy; however, she agreed to a renal biopsy on the 28^th^ day of admission, with a serum creatinine level of 149.2 μmol/L. The light microscopy specimen contained 28 glomeruli, 5 of which were globally sclerotic, and other glomeruli presented with mild hyperplasia of mesangial cells and mesangial stroma, as well as oxalate crystallization in some renal tubular lumens and acute tubular interstitial injury with chronic transformation. The crystallization exhibited birefringence under a polarized light microscope (Fig. 2). Due to limited laboratory conditions, measurement of oxalate in the serum and urine was lacking. Prednisone (20 mg/day) was administered orally, along with daily chewable vitamin D and calcium tablets. On discharge, her serum creatinine was 139.0 μmol/L, and urine output was normal. The level of urinary microalbumin was 27.7 mg/L (0–30), that of urinary alpha1-microglobulin was 38.67 mg/L (0–18), and the urinary microalbumin/creatinine ratio was 27.6 mg/g. cr (0–30) 1 week after discharge. Serum creatinine showed a downward trend during the follow-up, and her creatinine had reduced to 116.8 μmol/L 1 month after discharge. Serum creatinine was 102.8 μmol/L and 24 h proteinuria was 187 mg/24 h at 3 months post-discharge. Proteinuria was 135 mg/24 h at 5 months post-discharge. Steroid therapy was continued for approximately 6 months with gradual tapering. Steroids, however, seemed superfluous. The serum creatinine level was 92.8 μmol/L (41–81) and 24 h proteinuria was 113 mg/24 h at 20 months post-discharge.

### Case 2

A 60-year-old woman was referred to our nephrology department owing to abdominal distension, vomiting, and chest tightness. She had a history of hypertension and consumption of nonsteroidal anti-inflammatory drugs for rheumatoid arthritis for the last 10 years. The patient had no history of nephropathy, and her baseline serum creatinine was unknown. ​She had not taken calcium supplements prior to admission. Upon arrival, her serum creatinine soared to 844.1 μmol/L, with hemoglobin levels of 111 g/L. Anti-dsDNA antibody, anti-Sm antibody, MPO-ANCA, PR3-ANCA, and anti-GBM antibodies were negative. Urine test results were as follows: occult blood (-), protein (-), crystallization (-), urine specific gravity 1.008, and 24-h urine protein 316 mg/24 h. Renal ultrasound showed normal-sized kidneys (left kidney, 115 × 59 × 57 mm; right kidney, 109 × 57 × 55 mm). No abnormalities were found in the ureters and bladder. After intravenous fluid replacement, the urine volume was 750 mL and daily hemodialysis was performed from the second day of hospitalization for a total of five sessions. The patient’s urinary output increased to 1400 mL on the third day after admission. Unfortunately, a kidney biopsy was not performed because the patient had been taking aspirin for coronary heart disease, and a routine chest scan revealed a solid mass in the left upper lobe and sputum smear showed *Mycobacterium tuberculosis* bacilli. Serum creatinine level decreased to 284 μmol/L on the 7^th^ day of hospitalization, the dialysis was discontinued, and the patient was discharged for further treatment of pulmonary tuberculosis. We inquired about her diet in detail, and approximately 1 kg of purslane had been ingested (0.5 kg each over two consecutive meals) nine days before admission. Therefore, our presumed diagnosis was oxalate nephropathy leading to AKI. Three weeks after hospital discharge, her creatinine was 96 μmol/L.

## Discussion and conclusions

Toxic nephropathy may present with acute tubular necrosis, acute interstitial nephritis, chronic interstitial fibrosis, and urinary tract carcinoma, as well as crystalluria and nephrolithiasis [1].​ Herbs are popular worldwide, and the number of patients with impaired health due to unregulated herbal therapy has increased. Herb toxicity may be intrinsic or secondary to the presence of undisclosed drugs or heavy metals, active compounds, herb-drug interactions, or incorrect identification [5]. Herbal nephropathy has been reported in various types of nephropathy, and the mechanisms of kidney injury include tubular cell toxicity, inflammation, and crystal nephropathy [1].

Purslane has been used as a medicinal herb and traditional food globally since time immemorial. Purslane belongs to the *Portulacaceae* family, a small family with 21 genera and 580 species. It is widely distributed worldwide, and mainly flourishes in the tropics and subtropics [3]. In Central Europe, Asia, and the Mediterranean, purslane is popular as a potherb. The soft stems and leaves can be eaten raw, alone, or with other vegetables, and are also used in cooking or as a salad. Oxalate and citric acid are the most abundant organic acids in purslane [6]. The oxalate content of purslane leaves is reported as 671–869 mg/100 g of fresh weight [2], compared with 658 and 1090 for spinach and amaranth per 100 g serving, respectively [7]. It also contains 26.6 mg of ascorbic acid per 100 g of fresh weight [2].

In the human body, oxalate is mainly derived from hepatic synthesis [8], followed by intestinal sources. In addition, ascorbic acid/vitamin C can be broken down to oxalate [9]. Oxalate is mainly absorbed in the colon and can be freely filtered through the glomerulus and is excreted via SLC26A6 expressed on the apical membrane of the renal proximal tubular epithelium [10]. Hyperoxaluria is defined as urinary oxalate excretion of > 40–45 mg (0.45–0.5 mmol) per day [7]. Hyperoxaluria can be divided into primary and secondary. The diagnosis of primary hyperoxaluria is confirmed by genetic testing [11]. Primary hyperoxaluria is an autosomal recessive genetic disease characterized by urinary oxalate excretions often higher than 100 mg/day [7] and is more common in children, with a median age at onset of 5.5 years [12]. Primary hyperoxaluria has three types (types 1–3) with various enzyme deficiencies in the glyoxylate metabolism pathway that causes excessive production of glyoxylate in the liver. Excessive oxalate deposition can lead to multiple organ involvement, including retinopathy (macular crystallization, retinal edema, and optic disc atrophy), heart diseases (cardiac conduction blocks and cardiomyopathy), kidney injury (oxalate nephropathy), osteopathy (pathological fractures and skeletal deformity), skin injury, and neuropathy [11]. Approximately 70% of the primary hyperoxalurias are type 1 (deficiency of alanine-glyoxylate aminotransferase (AGT) due to mutations in AGXT), 10% are type 2 (deficiency of glyoxylate reductase due to mutations in *GRHPR*), and 10% are type 3 (deficiency of 4-hydroxy-2-oxo-glutarate aldolase due to mutations in *HOGA1*) [12–14]. Hyperoxaluria can lead to calcium oxalate kidney stones, renal calcinosis, and progressive renal injury in types 1 and 2 [15, 16]. Renal failure is rare, but recurrent calcium oxalate stones are common in type 3, and renal function appears better maintained in patients with type 3 compared to that of patients with type 1 or 2 [13, 17]. Secondary hyperoxaluria is most commonly caused by enterogenic etiologies, and intestinal fat malabsorption for various reasons, such as short bowel syndrome, Roux-N-y intestinal bypass surgery, chronic pancreatitis, chronic diarrheal disease, and use of orlistat therapy, results in reabsorption of free oxalate due to undigested fatty acids binding to calcium in the colon [18–21]. In addition, high dietary oxalate intake, such as spinach, amaranth, and other green leafy vegetables, can result in secondary hyperoxaluria, and dietary calcium deficiency can also lead to hyperoxaluria owing to a large amount of free oxalate reabsorption in the colon [22]. The application of antibiotics and inflammatory bowel disease could inhibit the growth of *Oxalobacter formigenes*, thereby reducing the degradation of intestinal oxalate and leading to increased oxalate absorption [23, 24].

Given the patient's diet and oxalate crystals from renal biopsy, diet-induced secondary hyperoxaluria was proposed. Diet-induced oxalate nephropathy is uncommon; however, cases of oxalate nephropathy due to excessive intake of spinach, nuts, and vitamin C have been reported in the literature [25]. The initial clinical presentation of these cases may be different, with some cases presenting with nausea, vomiting, diarrhea, and oliguria (as in our cases), others with only poor appetite [26], and some with weakness and altered mental status [27]. The absence of a family history, history of urinary stones, and systemic deposition of calcium oxalate did not support a diagnosis of primary hyperoxaluria. Oxalate intake varies by region. Humans ingest on average 15–25 mmol (600–1000 mg) of calcium and 1–3 mmol (90–270 mg) of dietary oxalate per day [28]. Most oxalates are excreted from the intestine as insoluble calcium oxalate, and urinary oxalate excretion is less than 0.50 mmol (45 mg) /1.73 m2 per day [11]. In one study, ​when dietary oxalate increased from 10 to 250 mg per day, the mean contribution to urinary oxalate ranged from 25 to 42% [29]. When urinary oxalate excretion increased from 20 to 40 mg per day, the relative risk of calcium oxalate stone disease increased 2.5–3.5 fold [30]. Secondary hyperoxaluria caused by dietary oxalate alone may occur in cases of extremely elevated oxalate intake (> 1000 mg/day) [7]. Serum and urine oxalate concentrations are also elevated in oxalate nephropathy. Accurate measurements of oxalate concentrations in plasma and serum remain challenging due to the need for rapid acidification of blood samples [31]. The weight of purslane was estimated for our patients, and data on plasma or urine oxalate concentration at admission and serum oxalate concentration before and after dialysis were missing due to our laboratory limitations. Thus, we could not precisely evaluate the amount of oxalate ingested.

In addition to renal biopsy, evidence of calcium oxalate crystals on repeat examination of the urine sediment is an important diagnostic clue [32]. Unfortunately, calcium oxalate crystals were not found in the urine sediment of our patients. Our case highlights the importance of renal biopsy, characterized by birefringent oxalate crystals under a polarized light microscope. Oxalate forms insoluble crystals with calcium, and calcium oxalate is saturated within the distal end of the descending limb of the loop of Henle. Calcium oxalate crystals are commonly found in the proximal and distal tubules in the cortex [33]. Oxalate nephropathy is defined as renal tubular injury, interstitial fibrosis, or progressive renal impairment caused by calcium oxalate crystal deposition [34]. Oxalate nephropathy is a pathological diagnosis. Polarized light microscope shows birefringent crystals with hematoxylin and eosin staining [20]. Actually, calcium oxalate crystals show great variability in distribution and quantity, and the amount of crystalline deposits in renal tubules required to diagnose oxalate nephropathy has not been clearly determined. A recent review article suggested adding an oxalate crystal-to-glomerulus ratio of ≥ 0.25 in the definition of oxalate nephropathy [35]. Our patient in case 1 did not reach this proportion, which may be related to the obvious improvement of renal function at renal biopsy. Buysschaert et al. concluded that the morbidity of oxalate nephropathy is approximately 1% (22 cases) based on the screening of 2,265 kidney biopsies in a Belgian series from 2010 to 2018, and approximately half the patients progressed to renal failure during the 29 months of follow-up [36]. Calcium oxalate crystals can damage the tubular epithelium and induce apoptosis [37]. Renal tubular cells release proinflammatory factors, which recruit immunocytes to access the renal interstitium and activate dendritic cells and macrophages [38]. Calcium oxalate crystals impair kidney function by blocking the renal tubules, causing sterile inflammation and renal tubulointerstitial injury, and the nucleotide-binding domain, leucine-rich repeat inflammasome 3 (NLRP3) inflammasome activation plays an important role in causing interstitial fibrosis [39, 40]. Animal studies have shown that NLRP3-null mice were protected from renal insufficiency [39].

The treatment of hyperoxaluria includes etiological and symptomatic treatment. Conservative management of primary hyperoxaluria includes high fluid intake and the use of antagonists of calcium oxalate crystallization. Supplementation of pyridoxine is helpful in primary hyperoxaluria type 1, which is characterized by AGT enzyme deficiency, and pyridoxine is a cofactor of this enzyme. Hepato-renal transplantation is currently an effective treatment option, for primary hyperoxaluria type 1 [12]. Primary hyperoxaluria type 2 requires only a kidney transplant. Furthermore, kidney failure is rare in primary hyperoxaluria type 3; therefore, a kidney transplant is generally not required. New promising therapies are being developed, such as RNA interference therapies, lumasiran, and nedosiran [41, 42]. The underlying etiological treatment is important for secondary hyperoxaluria and includes avoidance of high dietary oxalate intake and improvement of fat malabsorption. The treatment to reduce urinary oxalate concentration includes a low-oxalate diet, high fluid intake, ingestion of calcium supplements, and use of crystallization inhibitors, such as sodium potassium or sodium citrate [43]. Steroid administration in either primary or secondary hyperoxaluria is not necessary for oxalate nephropathy.

Our patients developed AKI after ingestion of purslane, and a similar case has been reported recently [44]. In case 1, mild glomerular changes in renal biopsy may be consistent with microalbuminuria that was insufficient to affect renal function, and the urinary microalbumin/creatinine ratio was 27.6 mg/g 1 week after discharge, but the creatinine level did not fully return to normal during the follow-up. In case 2, we hypothesized the diagnosis of oxalate nephropathy after excluding other possibilities, without renal biopsy support. Their baseline creatinine level prior to onset was unclear, thus they were more likely to have pre-existing chronic kidney disease, and at least a part of the renal dysfunction could be based on those conditions, and they had risk factors including older age, hypertension, and diabetes. A previous study showed that patients with diabetes have higher urinary oxalate concentrations than those of healthy controls [45], and patients with diabetes are more likely to develop hyperoxaluria. Acute dehydration due to vomiting may also be a contributing factor to increased urinary oxalate concentration.

Regarding treatment interventions, withdrawal of oxalate-rich foods or precursors after the occurrence of AKI, and adequate fluid intake and calcium supplementation, are necessary. As calcium intake is low, urinary oxalate excretion increases. A low dietary oxalate intake < 100 mg/day and adequate dietary calcium intake of 1000–1200 mg/day are recommended [46]. In addition, renal replacement therapy can be administered based on the patient's actual condition. However, some cases require only conservative treatment [27]. We also prescribed steroids for the patient in Case 1 to suppress tubular inflammation. However, steroids appear redundant, and to our knowledge, there are no reports in the literature indicating their necessity. Furthermore, the prognosis of oxalate nephropathy is variable and may be favorable in cases of acute intake of excessive dietary oxalate. Our patients required temporary hemodialysis and had almost complete renal recovery.

The possibility of oxalate nephropathy should be considered in the presence of calcium oxalate stones, calcium oxalate crystals in urine sediment, unexplained renal damage associated with multicentric systemic oxalosis, and calcium oxalate crystallization by renal biopsy. The physician should correlate these features with the patient’s clinical details, including history of gastrointestinal surgery, dietary status, medication history, and genetic history. A renal biopsy should also be actively performed if there are no contraindications, even when genetic testing is performed to exclude primary hyperoxaluria, particularly in children. Rapid diagnosis and treatment may prevent progressive renal failure caused by oxalate crystal deposition in the kidney. Ultimately, nephrologists should be aware of the potential nephrotoxicity and safety profiles of various herbs and edible plants to help save lives, especially in chronically ill patients.

## Data Availability

All the data supporting this case report is contained within the manuscript.

## Abbreviations

AKI: Acute kidney injury
DM: Diabetes mellitus
MPO-ANCA: Myeloperoxidase-antineutrophil cytoplasmic antibody
PR3-ANCA: Proteinase 3 antineutrophil cytoplasmic autoantibody
Anti-GBM: Anti-glomerular basement membrane
AGT: Alanine-glyoxylate aminotransferase
